# Epithelial Hypusination Regulates Helicobacter pylori-induced Gastric Inflammation

**DOI:** 10.21203/rs.3.rs-7906468/v1

**Published:** 2025-11-05

**Authors:** Alain P. Gobert, Kara M. McNamara, Caroline V. Hawkins, Mohammad Asim, Daniel P. Barry, Alberto G. Delgado, Kristie L. Rose, Purvi Patel, Regina N. Tyree, Kate S. Carson, Lori A. Coburn, M. Blanca Piazuelo, Keith T. Wilson

**Affiliations:** Vanderbilt University Medical Center; Vanderbilt University Medical Center; Vanderbilt University Medical Center; Vanderbilt University Medical Center; Vanderbilt University Medical Center; Vanderbilt University Medical Center; Vanderbilt University School of Medicine; Vanderbilt University School of Medicine; Vanderbilt University Medical Center; Vanderbilt University Medical Center; Vanderbilt University Medical Center; Vanderbilt University Medical Center; Vanderbilt University Medical Center

**Keywords:** Infection, Gastritis, Polyamines, Hypusine, Proteome

## Abstract

Hypusine is a unique amino acid synthesized on the eukaryotic initiation factor 5A (EIF5A) from the polyamine spermidine by deoxyhypusine synthase (DHPS). Hypusination of EIF5A plays a key role in translation. Here, we examined the contribution of the epithelial hypusination pathway to gastric inflammation induced by *Helicobacter pylori*. Immunohistochemical analyses revealed increased expression of DHPS and hypusinated EIF5A (EIF5A^Hyp^) in the gastric mucosa of patients with *H. pylori* gastritis compared to uninfected individuals, notably within gastric epithelial cells (GECs) and immune infiltrates. Then, we created a mouse model with epithelial-specific deletion of Dhps (*Dhps*^*Δepi*^) and confirmed the reduction of DHPS and EIF5A^Hyp^ in GECs. *H. pylori*-infected *Dhps*^*Δepi*^ mice exhibited an attenuation of gastric histologic inflammation scores compared with infected *Dhps*^*fl/+*^ controls, without alteration in bacterial colonization levels. Quantitative proteomics of isolated GECs showed that *Dhps* deletion altered the expression of proteins involved in organismal injury, cancer, and gastrointestinal diseases in naïve mice. Upon *H. pylori* infection, inflammatory and immune response proteins, including signaling factors and immunoglobulin mediators, were less induced in *Dhps*^*Δepi*^ GECs, and pathways linked to tissue injury and inflammation were selectively downregulated. Together, these findings demonstrate that epithelial hypusination supports *H. pylori*-driven gastric inflammation without affecting bacterial persistence. Targeting DHPS-dependent EIF5A hypusination may thus represent a novel therapeutic strategy to limit *H. pylori*-associated mucosal injury and disease progression.

## Introduction

The polyamine spermidine serves an essential role as the substrate for the synthesis of hypusine, a unique amino acid found only in the highly conserved eukaryotic protein eukaryotic translation initiation factor 5A (EIF5A) (Park et al. 1981). The hypusine modification is catalyzed by the sequential action of two enzymes, deoxyhypusine synthase (DHPS), the rate-limiting enzyme, which transfers the 4-aminobutyl moiety of the polyamine spermidine to the Lys50 residue of EIF5A (Joe et al. 1995; Park et al. 2006), and deoxyhypusine hydroxylase (DOHH), which hydroxylases the intermediate deoxyhypusine residue (Abbruzzese et al. 1986). This post-translational modification is essential for the function of EIF5A, as hypusinated EIF5A (EIF5A^Hyp^) can bind mRNAs that contain a 5′-AAAUGU-3′ consensus sequence (Maier et al. 2010; Xu et al. 2004). EIF5A^Hyp^ can also alleviate ribosome stalling at polyproline repeats during translation elongation and at other non-polyproline motifs, such as peptides enriched in basic amino acids (Pelechano and Alepuz 2017; Schuller et al. 2017).

Hypusination has been implicated in regulating inflammatory responses, specifically in myeloid and T cells (Gobert et al. 2020; Puleston et al. 2021). We have reported that hypusination in myeloid cells supports the antimicrobial response of macrophages to pathogenic bacteria including *Helicobacter pylori* (Gobert et al. 2020), a bacteria that colonizes the human stomach and causes diseases ranging from non-atrophic gastritis to the precancerous lesions of multifocal atrophic gastritis, intestinal metaplasia (IM), dysplasia, and gastric adenocarcinoma (Correa 1988; Piazuelo et al. 2021). Notably, mice with a myeloid-specific deletion of *Dhps* exhibited increased bacterial burden and inflammation, implicating hypusination in host defense against pathogenic bacteria (Gobert et al. 2020). However, the effect of hypusination in gastric epithelial cells (GECs) on *H. pylori* pathogenesis remains unknown.

In this report, we showed that patients with *H. pylori* gastritis exhibit increased level of DHPS and hypusinated EIF5A. We then created mice with specific deletion of *Dhps* in intestinal epithelial cells including in the stomach to assess the role of hypusination in GECs in *H. pylori* pathogenesis. We found that these infected mice develop less gastritis, demonstrating that the activity of DHPS in GECs supports stomach inflammation. Further, the proteome of the GECs in *Dhps*-deficient mice is reshaped toward a less inflammatory and carcinogenic profile.

## Materials and methods

### Ethics statement

Endoscopic gastric biopsies were obtained from patients at the Nashville Veterans Affairs Medical Center of the VA Tennessee Valley Healthcare System. Patients were undergoing esophagogastroduodenoscopy for clinically indicated reasons and provided informed consent for obtaining research biopsies under VA IRB protocol 1571167.

The mice were used under protocols V2000018 and V2300022 approved by the Vanderbilt University Medical Center Institutional Animal Care and Use Committee and the Research and Development Committee of the Veterans Affairs Tennessee Valley Healthcare System. Procedures followed institutional policies, AAALAC guidelines, the AVMA Guidelines on Euthanasia, NIH regulations regarding the Guide for the Care and Use of Laboratory Animals, and the United States Animal Welfare Act of 1996.

### Bacteria

*H. pylori* PMSS1, a *cagA*^*+*^ strain with intact type IV secretion system function, was grown on Trypticase soy agar plates containing 10% sheep’s blood. Bacteria were harvested from the plates and grown overnight in Brucella broth containing 10% fetal bovine serum (FBS). This culture was resuspended in fresh Brucella broth-FBS and then collected at the exponential phase to infect the mice.

### Mice and infections

We used *Foxa3*-cre mice that we crossed with *Dhps*^*fl/fl*^ mice to obtain C57BL/6 *Dhps*^*fl/+*^;Foxa3^+/+^ (*Dhps*^*fl/+*^) and *Dhps*^*fl/+*^;*Foxa3*^*cre/+*^ (*Dhps*^*Δepi*^) mice. Note that *Dhps*^*fl/fl*^; *Foxa3*^*cre/+*^ mice were embryonically lethal or only survived a few weeks out of utero. Mice were housed in a pathogen-free facility, with ventilated cage racks and were on a 12 h light-dark cycle. Male and female mice between 8 and 12 weeks were used for all studies. Animals were infected by oral gavage with 10^9^ colony forming units (CFU) of *H. pylori* PMSS1 in 200 μL Brucella broth, two times, on days 0 and 2. The control mice were gavaged with only broth on both days. Eight weeks after the first infection, mice were euthanized and stomachs were harvested. Colonization was determined in all infected mice by counting the CFUs cultured after plating serial dilutions of homogenized gastric tissues (Latour et al. 2022; McNamara et al. 2025; Sierra et al. 2020).

### Histopathology

Human biopsies from the gastric antrum and corpus, and longitudinal strips of murine stomach tissue including the corpus and antrum, were fixed in 10% neutral buffered formalin, paraffin-embedded, and stained with hematoxylin and eosin (H&E). Biopsies were scored as reported (Latour et al. 2022; McNamara et al. 2025; Sierra et al. 2020). Histology was scored by our gastrointestinal pathologist (M.B.P.) who was blinded to the experimental groups. H. pylori infection of patient tissues was confirmed by culture of gastric biopsies as described above for the mouse tissues.

### Epithelial cell isolation

Stomachs were removed from *Dhps*^*fl/+*^ and *Dhps*^*Δepi*^ animals and incubated in a solution of cold 0.5 mM DTT and 3 mM EDTA for 30 min on ice. After incubation, the tissues were placed in 3 mM EDTA and vigorously shaken to release gastric glands. The cell suspension was poured over a 70 μm Strainer (Falcon) and the resulting isolated GECs were pelleted through centrifugation at 1500 rpm for 10 min at 4 °C.

### Proteomics analysis

Isolated epithelial cells were lysed in 50 mM Tris-HCl pH 7.6, 150 mM NaCl, 1% NP-40, 2 mM EDTA, and 1% SDS; protein concentration was measured by the BCA Protein Assay (Pierce) and samples from the same group were pooled. Protein extracts were reduced with 10 mM TCEP (tris(2-carboxyethyl)phosphine), alkylated with 20 mM iodoacetamide, and protein samples were prepared by S-Trap^™^ (ProtiFi) digestion with trypsin (1:10) similar to methods described in Howard *et al* (Howard et al. 2024). TMT-based quantitative proteomics was performed as described (Latour et al. 2022). Labeled peptides (5 μg per sample) were combined, fractionated using high pH reversed phase fractionation, and elution steps were performed with 10%, 12.5%, 15%, 17.5%, 20%, 22.5%, 25%, and 60% acetonitrile with 0.1% triethylamine. Fractions were dried and reconstituted in 0.2% formic acid for LC-MS/MS analysis. Peptides were gradient-eluted at a flow rate of 350 nl/min, using varied reverse phase gradients over 90 min. For fraction 1, peptides were analyzed with the following gradient: 5–18% B in 75 min, 18–50% B in 6 min, 50–70% B in 3 min, 70 − 2% B in 1 min, 2% B for 5 min. For fractions 2–4, the first 2 steps of the gradient were adjusted to 5–25% B in 75 min and 25–50% B in 6 min, with the subsequent three steps identical to fraction 1. For fraction 5, the gradient included 2–8% B in 0.5 min, 8–30% B in 74.5 min, 30–50% B in 6 min, 50–70% B in 2 min, followed by the same final two steps. For fraction 6, the gradient included 2–8% B in 2 min, 8–30% B in 73 min, 30–50% B in 7 min, 50–70%B in 1 min, followed by the same final two steps. For fraction 7–8, the gradient included 5–45% B in 75 min, 45–90% B in 8 min, 90% B for 1 min, 90 − 2% B in 1 min, and 2% B for 5 min. Peptides were analyzed using a data-dependent acquisition method on an Orbitrap Exploris 240 mass spectrometer (Thermo Scientific), equipped with a nanoelectrospray ionization source. The instrument method consisted of MS1, followed by up to 20 MS/MS scans, with an automatic gain control target of 2×10^5^. Higher-energy collisional dissociation was set to 35 nce and dynamic exclusion (15 sec) was enabled. Data were searched in Proteome Discoverer 2.2 (Thermo Scientific) using SequestHT for database searching against a mouse database created from the UniProtKB database. Search parameters and quantitative analysis was performed as reported (Latour et al. 2022).

The mass spectrometry proteomics data have been deposited to the ProteomeXchange Consortium via the PRIDE (Perez-Riverol et al. 2025) partner repository with the dataset identifier PXD069622

Ingenuity pathway analysis (IPA) software (QIAGEN) was used for the functional interpretation of differential expression results obtained from the proteomic analyses. The pathways related to Diseases and Functions were generated.

### Western blot analysis

#### Western blot analysis

Proteins were extracted from isolated GECs as reported (Latour et al. 2022) and concentrations were determined using the BCA Protein Assay (Pierce). Western blots were performed using 10 μg protein per lane using a rabbit polyclonal anti-DHPS antibody (Ab; Abcam, Cat#ab202133; 1:5000), a rabbit polyclonal anti-EIF5A^Hyp^ Ab (Millipore, Cat#ABS1064-I; 1:8000), or a mouse monoclonal anti-b-actin Ab (MilliporeSigma, Cat#A5316; 1:10000). The Peroxidase AffiniPure^®^ Goat Anti-Rabbit IgG (H + L) (Jackson ImmunoResearch, Cat#111-035-003; 1:5000) or the goat anti-mouse IgG, HRP-labeled (Jackson ImmunoResearch, Cat#115-035-003; 1:5000) were the used as secondary Abs.

### Immunostaining

Immunofluorescence was performed on human gastric biopsies and murine gastric tissues. Sections were deparaffinized and incubated at room temperature with 3% hydrogen peroxide in phosphate-buffered saline to block endogenous peroxidase. Tissues were then blocked for 1 h in Protein Block, Serum-Free (Dako, Cat#X0909). Slides were sequentially incubated with a rabbit polyclonal anti-DHPS Ab (Proteintech, Cat#11184–1-AP; 1:1000) or a rabbit anti-EIF5A^Hyp^ Ab (MilliporeSigma Cat#ABS1064; 1:2000) overnight at 4°C and with a donkey anti-Rabbit IgG (H + L) Highly Cross-Adsorbed Secondary Antibody, Alexa Fluor^™^ Plus 488 (ThermoFisher Scientific, Cat#A32790; 1:700) 45 min at room temperature. Slides were mounted with VECTASHIELD HardSet^™^ Antifade Mounting Medium with DAPI (Vector Laboratories, Cat#H-1500–10) and visualized using a Nikon E800 microscope and a SPOT Imaging CMOS camera.

### Statistics

Prism 10.6.0 (GraphPad Inc.) was used for figure design and statistical analysis. All the data are expressed as mean ± SEM. Data that were not normally distributed according to the D’Agostino & Pearson normality test were log transformed. Student’s *t* test was used to determine significant differences between two groups, whereas a one-way ANOVA followed by a Tukey’s test or Šídák’s test was used for multiple groups.

## Results

### Increased levels of DHPS and EIF5A^Hyp^ in H. pylori-infected patients

**Figure 1:**
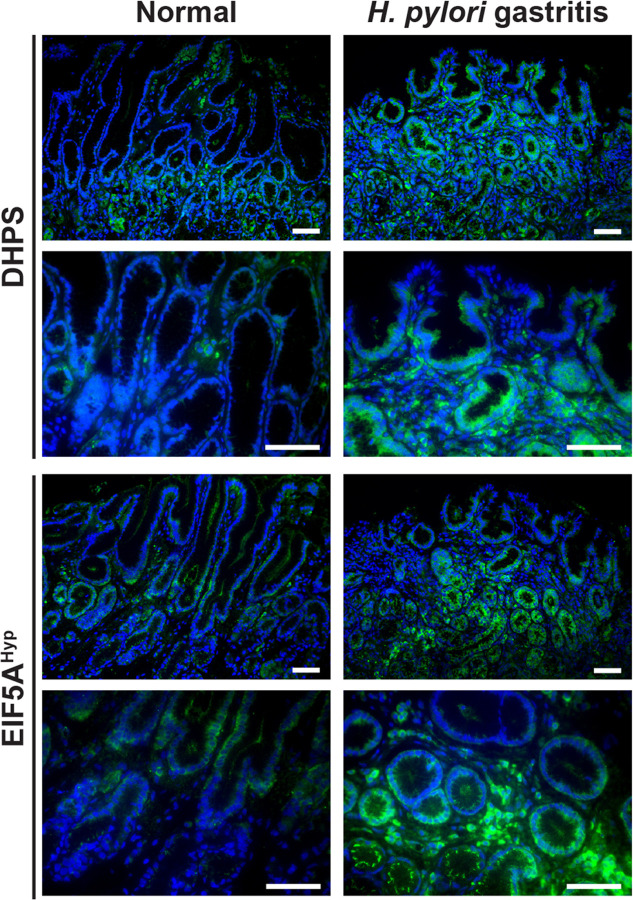
Levels of DHPS and EIF5A^Hyp^ in patients infected with *H. pylori*. Gastric biopsies were obtained from patients with gastritis and *H. pylori* infection, determined by histology and culture of ground tissues, and from normal individuals. Tissues were immunostained for DHPS or EIF5A^Hyp^ (green); nucleus were stained with DAPI and appear in blue. These data are representative immunofluorescence images of n = 3–4 patients per group.

Using immunostaining, we evidenced that that the level of DHPS and hypusinated EIF5A were increased overall in the gastric mucosa of endoscopic biopsies from patients with *H. pylori* gastritis compared to individuals without infection (Fig. 1). Of importance, the staining was abundant in GECs in *H. pylori*-infected patients but also present in the immune infiltrates, as we reported (Gobert et al. 2020).

### Deletion of epithelial hypusination reduces the inflammatory response to H. pylori

**Figure 2:**
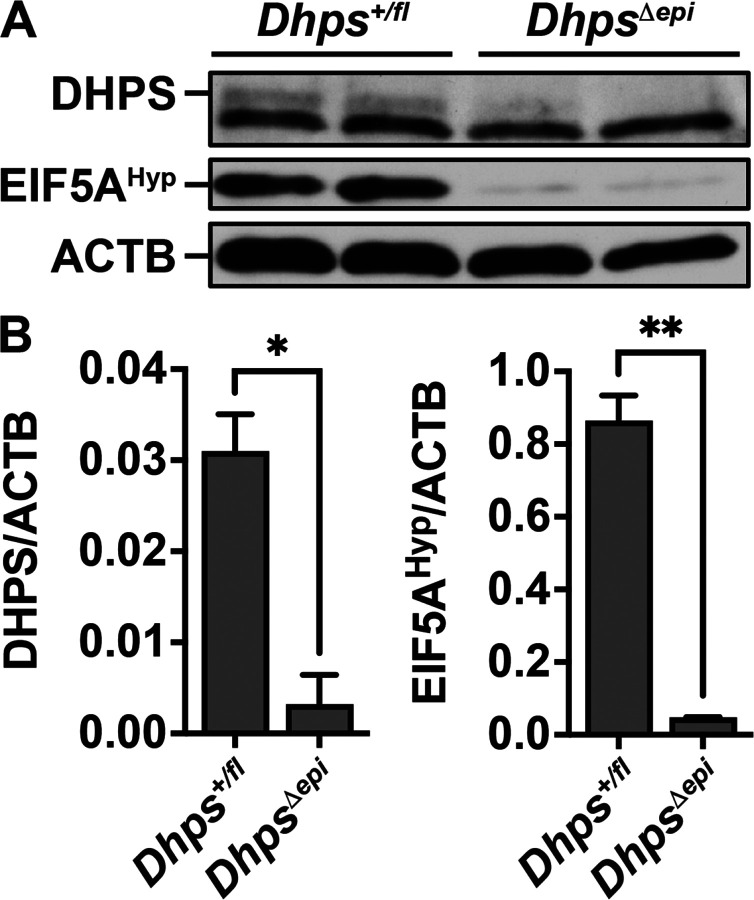
Loss of DHPS and hypusination in *Dhps*^*Depi*^ mice. GECs were isolated from the stomach of *Dhps*^*fl/+*^ and *Dhps*^*Depi*^ mice. Proteins were extracted and the levels of DHPS and EIF5A^Hyp^ were determined by Western blots (**A**) and densitometry (**B**).

To investigate the role of hypusination in epithelial cells during *H. pylori* infection we utilized a genetic approach by generating C57BL/6 animals with a gastric epithelial-specific knockout of *Dhps*. First, we verified by immunoblots and densitometry that *Dhps*^*Δepi*^ mice exhibited reduced expression of DHPS and EIF5A^Hyp^ levels in the gastric epithelium compared to *Dhps*^*fl/+*^ control mice (Fig. 2A and 2B).

**Figure 3:**
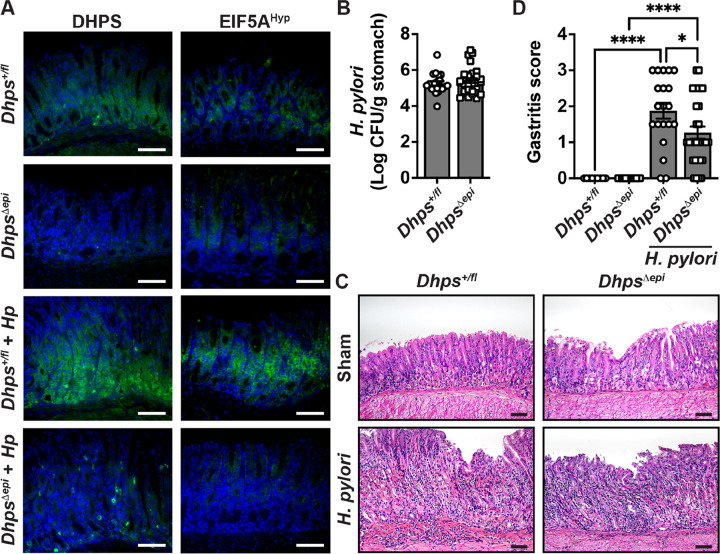
Effect of *Dhps* deletion in GECs on *H. pylori* pathogenesis. *Dhps*^*fl/+*^ (*n* = 21) and *Dhps*^*Depi*^ (*n* = 30) mice were infected or not with *H. pylori* PMSS1 for 8 weeks. (**A**) The presence of DHPS and EIF5A^Hyp^ in the gastric tissues was assessed by immunofluroresence (green); representative images of *n* = 5 mice per group are shown. (**B**) *H. pylori* colonization of gastric tissues was determined by culture of serial dilutions of homogenized tissues. (**C-D**) H&E images (**C**; scale bars: 50 μm) were used to establish the histologic gastritis scores (**D**); *P < 0.05 and ****P < 0.0001 by one-way ANOVA and Šídák’s test.

Then, we infected *Dhps*^*fl/+*^ and *Dhps*^*Δepi*^ mice with *H. pylori* PMSS1 for 8 weeks. We confirmed by immunofluorescence that DHPS and EIF5A^Hyp^ were less expressed in GECs from naive *Dhps*^*Δepi*^ mice (Fig. 3A). Upon infection, the levels of DHPS and EIF5A^Hyp^ were increased in GECs and immune infiltrates of *Dhps*^*fl/+*^ mice compared to uninfected animals (Fig. 3A); there was markedly less staining in the GECs of *Dhps*^*Δepi*^ mice, whereas immune cells were still positive for DHPS (Fig. 3A).

All 30 *Dhps*^*Δepi*^ mice were colonized and only 1 of the 21 *Dhps*^*fl/+*^ mice was not colonized and was thus removed from the analysis. We observed no difference in gastric bacterial burden between both genotypes (Fig. 3B). An inflammatory infiltrate and mild foveolar hyperplasia were mainly observed at the antrocorporal transitional mucosa of *H. pylori*-infected *Dhps*^*fl/+*^ mice (Fig. 3C). These parameters were less observed in the stomach of infected *Dhps*^*Δepi*^ mice (Fig. 3C). Using a comprehensive score, we found increased inflammation in both genotypes compared to uninfected animals, but also significantly less inflammation in the gastric tissue of infected *Dhps*^*Δepi*^ mice compared to the infected *Dhps*^*+/fl*^ mice (Fig. 3D).

### Proteome of H. pylori-infected animals with Dhps deficiency

To determine the role of the hypusination pathway during *H. pylori*-mediated inflammation, we performed TMT proteomics on isolated GECs from uninfected and infected *Dhps*^*fl/+*^ and *Dhps*^*Δepi*^ mice.

**Figure 4:**
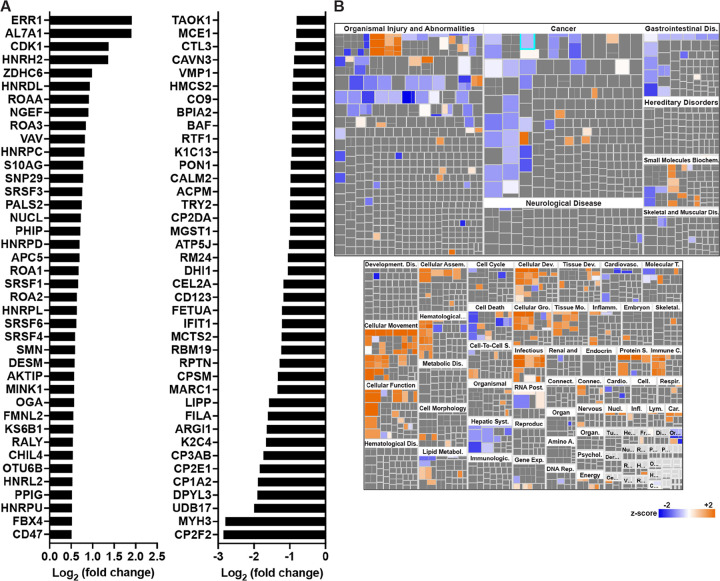
Proteomic changes in GECs of *Dhps*^*Depi*^ mice. (**A**) Proteins from isolated GECs of uninfected *Dhps*^*fl/+*^ and *Dhps*^*Depi*^ mice were analyzed by TMT and the 40 proteins that were the most significantly upregulated or downregulated in *Dhps*^*Depi*^ GECs are shown (*n* = 2 per group). The complete list of proteins identified is provided in Supplementary Table S1. (**B**) The differential proteomic dataset comparing GECs of *Dhps*^*Depi*^ to *Dhps*^*fl/+*^ mice was used to determine the “Disease & Functions” pathways using IPA. See Supplementary Table S1 for the complete list of pathways.

First, we analyzed the proteome in GECs from naïve mice. There were 79 proteins significantly induced by the specific *Dhps* deletion in epithelial cells (Supplementary Table S1). These proteins were signaling molecules, such as cyclin-dependent kinase 1 (CDK1) or Src substrate cortactin (SRC8), the superoxide dismutase SODM, and numerous heterogeneous nuclear ribonucleoproteins (ROA1/A2/A3/AA), which are RNA-binding proteins playing critical roles in multiple cellular processes such as DNA repair and regulation of gene expression (Fig. 4A and Supplementary Table S1). We also found 183 proteins downregulated in GECs from *Dhps*^*Δepi*^ mice. Among them we found numerous ribosomal proteins (e.g., RM24, RS4X, RT33), as expected, different cytochromes P450 (e.g. CP3AB, CP2E1, CP1A2), and one glutathione-S-transferase (MGST1) (Fig. 4A and Supplementary Table S1). The changes in the level of these proteins in GECs were overall associated with a lessening of the pathways associated with organismal injury, cancer, and gastrointestinal diseases in *Dhps*^*Δepi*^ mice (Fig. 4B and Supplementary Table S2); pathways associated with cell movement were mainly induced in *Dhps*^*Δepi*^ mice (Fig. 4B and Supplementary Table S2).

**Figure 5:**
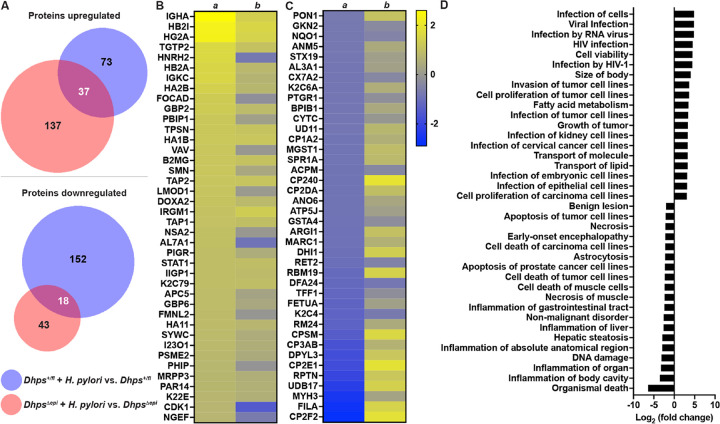
Regulation by hypusination of the proteome of GECs during *H. pylori* infection. *Dhps*^*fl/+*^ and *Dhps*^*Depi*^ mice were infected or not with *H. pylori* PMSS1 for 8 weeks. Proteins from isolated GECs were analyzed by TMT (*n* = 2 per group). (**A**) The number of proteins significantly upregulated or downregulated in infected mice compared to uninfected animals in both genotypes is depicted as a Venn diagram. (**B-C**) The heatmaps depict the level of expression of the 40 proteins that were the most significantly upregulated (**B**) or downregulated (**C**) in infected *Dhps*^*fl/+*^ compared to cells from uninfected mice (column *a*); the fold change in infected vs. uninfected *Dhps*^*Depi*^ GECs is shown in column *b*. (**D**) Functional analysis of the proteins differentially expressed in GECs from infected *Dhps*^*Depi*^ compared to infected *Dhps*^*fl/+*^ mice using IPA; the complete list of pathways related to “Disease & Functions” is provided in Supplementary Table S3.

We identified 110 and 174 proteins significantly induced by *H. pylori* infection in the GECs from the stomach tissues from *Dhps*^*fl/+*^ and *Dhps*^*Δepi*^ mice, respectively. Among them, 37 were in common to both genotypes (Fig. 5A). These included mediators of adaptive immunity (IGHA, HB2A/2I, HG2A, and TGTP2), and regulators of the innate immune response, such as DOXA2, I23O1, and STAT1 (Fig. 5B and Supplementary Table S1). However overall, the level of expression of these proteins in *H. pylori*-infected *Dhps*^*Δepi*^ mice was lower compared to infected *Dhps*^*fl/+*^ animals (Fig. 5B). Moreover, there were 170 proteins significantly downregulated in GECs from *Dhps*^*fl/+*^ mice with infection (Fig. 5A), whereas only 61 proteins were less expressed in infected *Dhps*^*Δepi*^ GECs (Fig. 5A); only 18 proteins were similar in both genotypes (Fig. 5A). The proteins downregulated by *H. pylori* infection in *Dhps*^*fl/+*^ animals included numerous cytochromes, e.g., CP2F, CP2E1, CP3AB, CP2DA, and CP240, which are known to be downregulated during infection and inflammation, TFF1, the stabilizer of the mucous gel overlying the gastrointestinal mucosa that provides a physical barrier against bacteria, and the marker of M2/Mreg macrophages, ARGI1 (Fig. 5C and Supplementary Table S1); most of these effectors were less altered in infected *Dhps*^*Δepi*^ mice (Fig. 5C and Supplementary Table S1).

When we analyzed the functional clusters corresponding to the proteins differentially expressed between infected *Dhps*^*Δepi*^ versus infected *Dhps*^*fl/+*^ mice (see Supplementary Table S1), we evidenced that numerous pathways related to infections were significantly upregulated, whereas the biological processes related to inflammation were mainly downregulated (Fig. 5D and Supplementary Table S3).

## Discussion

DHPS is the rate-limiting enzyme for the synthesis of hypusine on EIF5A, thus controlling its activation and the translation of specific proteins. In this report, we found that DHPS was induced in GECs of mice infected by the gastric pathogen *H. pylori*; consequently, the level of EIF5A^Hyp^ was also increased in the gastric epithelium. Interestingly, when we specifically knocked-down DHPS in intestinal epithelial cells, we found less *H. pylori*-induced gastritis in the stomach, suggesting that hypusination in GECs supports inflammation. Our proteomic investigation in isolated GECs from the mice confirmed that the reduction of hypusination was associated with reduced expression of proteins involved in pathophysiological processes in both naïve and infected mice. Lastly, the increased level of hypusination in GECs from patients with *H. pylori* gastritis underlines the clinical relevance of our findings and highlights DHPS as a potential target to reduce the development of the diseases associated with *H. pylori* infection.

The homozygous deletion of the *Dhps* gene results in embryonic lethality (Nishimura et al. 2012) and biallelic variants in the *DHPS* gene in humans have been linked to a neurodevelopmental disorder (Ganapathi et al. 2019), evidencing that DHPS activity is globally essential for embryogenesis and homeostasis, and therefore constitutively expressed. Notably, this gene can be induced, as we reported increased DHPS levels in macrophages infected with pathogenic bacteria, including *H. pylori* (Gobert et al. 2020), in human colonic epithelial cells (CECs) infected *in vitro* with enteropathogenic *Escherichia coli*, and in the colon of mice infected with *Citrobacter rodentium*, a bacterial pathogen of the colon that induces colitis in mice (Gobert et al. 2024). Moreover, the expression of DHPS is also increased in adipose tissue macrophages of obese mice and in bone marrow-derived macrophages from C57BL/6J mice stimulated toward an M1 phenotype with LPS + IFN-g (Anderson-Baucum et al. 2021). Similarly, we found increased expression of DHPS in GECs from *H. pylori*-infected humans and mice; this was associated with an enhanced level of EIF5A^Hyp^, as expected.

We previously reported that the specific deletion of DHPS in myeloid cells using a Lyz2-Cre driver yields an increased colonization of the colon by the rodent pathogen *C. rodentium* and of the stomach by *H. pylori* (Gobert et al. 2020). This observation led us to propose that hypusination supports macrophage activity, which was consistent with the loss of expression of innate proteins with antimicrobial functions in infected *Dhps*^*Dmye*^ mice (Gobert et al. 2020). Moreover, we have reported that mice with knock-down of *Dhps* in intestinal epithelial cells also exhibit increased *C. rodentium* burden in the colon (Gobert et al. 2024), although the hypusine-dependent proteome of macrophages differed from that of CECs in infected mice (Gobert et al. 2020; Gobert et al. 2024). Herein, we found that *Dhps* deletion in the stomach has no impact on gastric colonization by *H. pylori*, demonstrating that hypusination in GECs does not play a major role in the antimicrobial effect of the gastric mucosa and the cell-specificity of DHPS activity. Moreover, we found reduced inflammation and histological damage in *H. pylori*-infected *Dhps*^*Δepi*^ mice. In contrast, mice with specific *Dhps* deletion in intestinal epithelial cells exhibited spontaneous colitis and inflammation of the small intestine, increased susceptibility to dextran sulfate sodium-induced and *C. rodentium*-mediated colitis, and exacerbated tumorigenesis in response to the carcinogen azoxymethane (Gobert et al. 2024; Gobert et al. 2023) compared to *Dhps*^*fl/fl*^ animals. In this context, we propose that the role of hypusination in epithelial cells throughout the gastrointestinal tract is clearly organ specific, being protective in the colon and deleterious in the stomach. The reason behind this discrepancy is likely related to the nature of the transcriptomes of GECs and CECs that are drastically different due to their distinct physiological functions and microenvironments; therefore, the proteins regulated by hypusination in these organs are different, as we have observed in our previous report (Gobert et al. 2023) and in the present study.

DHPS activity is also controlled by the availability of its substrate spermidine (Gobert et al. 2023). The concentration of this polyamine is regulated by the enzyme spermine oxidase (SMOX) in the gastrointestinal tract (Gobert et al. 2022). Interestingly, we reported that deletion of *Smox* in C57BL6 mice and in cancer-prone transgenic FVB/N mice overexpressing the human gastrin gene reduces the development of gastritis and gastric carcinoma, respectively (McNamara et al. 2025; Sierra et al. 2020), demonstrating that SMOX activity mediates *H. pylori* pathogenesis. Although we attributed the deleterious effects of SMOX on the synthesis of the monocarbonyl electrophile acrolein in the stomach (McNamara et al. 2025), it is also possible that DHPS activity can be enhanced by the generation of spermidine by SMOX. Thus, collectively these data indicate that the spermidine/hypusine pathway is a critical mediator of *H. pylori* pathogenesis.

The development of precancerous lesions in *H. pylori*-infected patients often occurs in the context of chronic gastritis (Correa 1988; Piazuelo et al. 2021). Moreover, eradication of *H. pylori* does not necessarily reduce cancer risk once precancerous lesions are present (Ma et al. 2012; Mera et al. 2005). Therefore, the inhibition of hypusination in the stomach might represent a therapeutic approach to dampen gastritis, but also a preventive strategy to reduce the risk of gastric cancer development. Further, it has been reported that hypusination supports the growth and proliferation of various established cancer cell lines (Bandino et al. 2014; Fang et al. 2018; Zhao et al. 2025), including from the gastrointestinal tract (Coni et al. 2020). In this context, the study of the role of the spermidine/hypusine pathway on gastric cancer cells is warranted and is underway in our laboratory.

## Supplementary Material

Supplementary Files

This is a list of supplementary files associated with this preprint. Click to download.

- DhpsDgecTableS3.xls
- DhpsDgecTableS2.xls
- DhpsDgecTableS1.xlsx

## Data Availability

The mass spectrometry proteomics data have been deposited to the ProteomeXchange Consortium via the PRIDE (Perez-Riverol et al. 2025) partner repository with the dataset identifier PXD069622.
